# Miscellaneous

**Published:** 1897-09

**Authors:** 


### MISCELLANEOUS.

### A CONTRIBUTION TO THE THERAPEUTICS OF IRON.

The skeptical assertions of Dr. Bunge, regarding the value of ferruginous medication, at the Congress for Internal Medicine of

1895, evoked an almost unanimous and vigorous opposition in the discussion which followed the reading of his paper. The doubts expressed by him in reference to an insufficient absorption of the inorganic preparations of iron could at that time only be controverted, in the main, by the results of practical experience derived from the administration of iron. However, Quincke even then pointed out that his investigations on the subject, which had not yet been concluded, had demonstrated the absorption of iron preparations given for medicinal purposes, and their utilization in the body. In 1896, at the Congress for Internal Medicine, Quincke reported the results of his experiments which meanwhile had been completed, and which confirmed in every respect the above-mentioned statement. He had made it his aim to trace the course of iron along the intestinal canal, by means of micro-chemical reactions, and for this purpose fed white mice for a number of days with cheese, to which had been added various ferruginous preparations. The animals were killed during feeding, or after the lapse of a certain interval, and the viscera, especially the intestinal canal, hardened in alcohol, cut open and examined for the presence of iron with sulphide of ammonium as a reagent. It was thus found that iron is absorbed exclusively in the duodenum, and this applies both to the iron in the food and that administered medicinally. It was detected in the duodenal epithelium and in the stroma of the villi, and is visible even to the naked eye. Furthermore, iron is found deposited especially in the liver cells, in a form perceptible on microscopical examination, and in rare cases could be detected by microscopical means in the cortical tubules of the kidneys.

These investigations of Quincke have demonstrated incontestably that the favorable results which have been obtained, since olden times, from the administration of iron are actually attributable to its absorption, and not, as Bunge would have it, to accidental circumstances, to diet alone, or even suggestion. Control experiments in this direction with indifferent medicaments are readily carried out, and were repeatedly mentioned at the Congress of 1895. It should be added that these control experiments were followed by no change, or only by a transient improvement in the condition of the patient.

At the last Congress for Internal Medicine, the subject of the therapeutics of iron was so thoroughly ventilated by the foremost clinicians, as well as by numerous physicians in late years, that a new contribution would appear superfluous. This subject, however, is of such immense importance to the general practitioner, that a cumulation of material is necessary in order to eliminate the least doubt as to the efficacy of a therapeutic measure which, originating at first on the basis of speculation, and later supported by the results of empirical observations, has finally been demonstrated to be of value by exact experimentation.

In the following I will only discuss the clinical aspects of this question. I was encouraged in undertaking this work by my honored teacher, Dr. Mackenrodt, who has assisted me in every possible way. In the management of chlorosis and anemia, and the host of sequelæ of these diseases, the physician would be powerless if he had not in iron a specific, or at least a potent and indispensable adjunct to his other therapeutic resources. The patients, who belong for the most part to the working classes, give in the main the same group of symptoms: amenorrhea, scanty or profuse, weakening, irregular, usually premature, menses; headache, anorexia and dyspepsia; neuralgias, and almost invariably marked lassitude, which interferes markedly with their ability to work. In these cases prompt and radical help must be afforded, in order to restore to the patients their full working capacity as soon as possible. It is well known that the therapeutic value of the various iron preparations differs greatly. This is shown *a priori* by the abundance of manufactured products of this kind. My experience relates chiefly to three preparations, pilulæ chinini cum ferro, formula magistralis of Berlin, liquor ferri albuminati, and the neutral Pepto-Mangan (Gude). My results with the first of these three remedies have been very indifferent, while with the liquor ferri albuminati of the pharmacopœia they were somewhat better. I have instituted accurate examinations, however, with only Gude's Pepto-Mangan, and the data given further on relate to this remedy alone. Owing to my limited experience with the many other preparations employed by various authors, I would not designate the Pepto-Mangan as a universal remedy, or as the only efficient preparation.

That dietetic treatment alone may be successful in anemic and chlorotic patients was laid down as a dictum by Immermann and Reinert at the Congress for Internal Medicine of 1895. It is natural to suppose that poor and ill-nourished presons would gain in strength under the influence of a proper and invigorating diet; nevertheless, after eight to fourteen days a cessation in the improvement occurs and the old disorders return. These authors, as well as Nothnagel and v. Ziemssen, consider an invigorating diet as only a valuable adjunct; both of the latter, moreover, regard rest in bed for several weeks as an important factor in the cure. Since several years Mackenrodt has also instituted a large series of observations of this kind, not yet published, in which, for purposes of control, he employed quantitative estimation of the hemoglobin. It was found by him that under the influence of hygienic and dietetic regulations alone the quantity of hemoglobin in the blood increased only at the commencement of treatment, and then only in a dilatory manner.

In the case of one of my patients I proceeded as follows: I prescribed Pepto-Mangan (Gude), one teaspoonful three times daily after meals, and regulated the diet in accordance with the directions furnished with the preparation. Sour and fatty foods, as well as raw fruits, are to be avoided under all circumstances. Fritsch advises, indeed, that the desire for acids manifested by chlorotics should be gratified. According to my experience, however, this craving for acids is to be regarded as a pathological condition of the alimentary tract, which is made worse by further supply of acids, but can be successfully overcome by an unstimulating diet. In cases where the social conditions in any way permitted, I allowed the patient to take a small glass of red wine three times daily, but never during a period of one hour before and after the administration of the medicament, in order to prevent the combination of the tannic acid contained in the wine with the iron. The use of potatoes was restricted as much as possible, at least, during the first four weeks. Furthermore, I resorted to the dietetic regulations customary in these cases, but changed them to advantage when, as so often happens, obstinate constipation was present, and obtained generally excellent results. In contrast to several authors who made it a practice to remove any existing dyspepsia before resorting to the use of iron, I have followed the method of v. Ziemssen and Baumler, of at once administering iron—unless the presence of a severe gastric affection, especially ulcer of the stomach, could be positively determined—and observed as early as the end of one or two weeks an increase of appetite and subsidence of the gastric disorder.

I would lay especial stress upon systematic exercise in the open air. I ordered the patients, who, with but two exceptions, were treated out of bed, to take a stroll at midday, at first of five to ten minutes' duration. At the end of three to four days they were allowed to remain outdoors for five to ten minutes longer.

After each walk they were advised to take off their corsets, put on their slippers, and rest for an hour on the sofa. Under this treatment, the lassitude invariably vanished after a time.

In the manner thus described I have treated in all about sixty patients. In twenty-four cases I instituted quantitative estimations of hemoglobin at regular intervals of three, five, or eight days. Under normal conditions the quantity of hemoglobin in woman amounts to 12.59 per cent. when estimated in comparison with the other constituents of the blood. Among my cases the lowest amount met with was in a single instance, 30 per cent. of the normal, that is to say, of the above 12.59 per cent. Next to this was the following case with 32 per cent. of the normal:

Miss W. G., twenty-two years old, seamstress, related that she had been under treatment for four years for chlorosis. Since the age of nineteen, her menses had been scanty, occurring before the usual time, and of three to eight days' duration. On September 26, 1895, a *remotio secundinarum* occurred after an abortion induced in the fourth month. At present she complains of darting pains in the upper portions of the lungs, headaches, and rapid loss of strength.

January 9, 1896, anemic appearance; physical examination, especially of lungs, negative. Quantity of hemoglobin 32 per cent. Ordered Pepto-Mangan, Gude, diet, etc.

January 13, 1896, considerable improvement of the general condition. Hemoglobin, 45 per cent.

January 17, since previous day, diarrhea, due to gross errors in diet, troublesome eructations. Ordered tinct. opii, 15 drops three times daily. Hemoglobin, 47 per cent.

January 21, improved after use of tinct. opii, no more gastric pains or eructations. Headaches have completely disappeared, lassitude less marked. Hemoglobin, 55 per cent.

January 31, condition unchanged, ceased menstruating on previous day, the flow having lasted five days.

February 8-28. Patient feels well and no longer complains of pains in the lungs. Appetite and bowels regular. Hemoglobin, constantly 55 per cent.

March 5, no change. Hemoglobin, 62 per cent.

March 11, hemoglobin, 68 per cent. March 27, 77½ per cent.

Unfortunately, as in most of these cases, the patient's visits ceased as soon as she felt entirely capable of going to work. As a matter of fact, the increase of hemoglobin in this case was tardy, as in four other cases in which the quantity at the beginning was 34, 35, 37, and 38 per cent. of the normal. In eighteen other instances in which the initial amount was higher, viz., 42-75 per cent. of the normal, progress was more rapid as a rule.

I also derived exceedingly favorable results from the use of Pepto-Mangan, Gude, in patients who came to us for operations after having been exhausted by protracted hemorrhages. Of course convalescence in such cases is delayed; the system recuperates but slowly from the double injury inflicted by the losses of blood and the operative intervention. Digestive disturbances are especially apt to be troublesome. In these cases ferruginous medication often produces remarkable improvement.

I cannot close this paper without calling attention to the beneficial influence exerted by Pepto-Mangan, Gude, in anemic neuralgias, and as an illustration of its effects in this class of cases add in brief the following history of a case.

Mrs. K., aged thirty-five years, very pale and ill-nourished, suffers from intercostal neuralgia on the left side.

January 30, 1896, quantity of hemoglobin, 68 per cent. of the normal.

February 5. In the meantime has suffered on two days with violent headaches. Intercostal neuralgia persists. Appetite good; no gastric disturbances. Hemoglobin, 69 per cent.

February 12, no longer troubled with headaches with exception of one attack of neuralgia in the area supplied by the left supra-orbital nerve. The paroxysms of pain on the left side of the chest have become less frequent. The lassitude has subsided. The mucous membranes are still anemic. On the whole, the patient feels better and more vigorous than before the commencement of treatment. Hemoglobin, 75 per cent.

February 18, considerable improvement of neuralgias; no headaches, nor digestive disturbances. General health improved. Menses appear earlier than previously, this being the second day of the flow. Hemoglobin, 73 per cent.

February 26. During the preceding days transient deterioration of her conditions owing to mental excitement. Menstrual period has been normal. Hemoglobin, not estimated.

March 2, patient no longer complains. Intercostal neuralgias have ceased to occur except on rare occasions. Hæmoglobin, 76 per cent. March 13, health good in general. Iron discontinued on account of gastric disturbances which are said to result from excitement. Ordered strict diet and iron to be resumed.

March 19, complete restoration of health. Hemoglobin, 82 per cent.

I think I am justified in asserting that in my therapeutic trials with Pepto-Mangan I obtained all that can be rationally demanded. And I further consider myself warranted in stating that in view of the unquestionable necessity of ferruginous medication in certain troublesome constitutional affections this preparation acts as a most efficient and useful auxiliary to our therapeutic efforts.—*Dr. Gellhorn, Therapeutische Monatshefte, May*, 1897.

---

### THE SPECIFIC ACTION OF QUININE IN MALARIA.

Dr. E. C. Register, Editor of the *Charlotte Medical Journal*, read a paper with this title before the North Carolina Medical Society.

After many years of study, both clinical and microscopical, the doctor arrives at the following conclusion in reference to the specific action of quinine in the continued forms of malarial fever. He says a malarial fever without complications will subside after the plasmodia of malaria disappears from the blood; that we have in quinine the means to completely eradicate malarial poison from the body; that malarial fever occurring in a previously healthy subject, and in the central United States, if at once recognized and properly treated, never ends in death; that it is speedily curable, never continues, provided the nature of the disease be recognized and appropriate treatment employed.

Dr. Register has made microscopical examinations of the blood of several hundred patients suffering with remittent malarial fever, and has studied closely and thoroughly the crescentic and ring-shaped bodies which he says are the forms of the parasite which is responsible for the continued types of this fever, and he finds that the reason quinine does not always affect these irregular forms of the poison, is on account of the usual defects in its administration. He contends that the drug is very imperfectly absorbed when given by the stomach, and when the patient has a temperature of over 102 degrees. He says that in cases of continued malarial fever, if distinct and well marked intermissions of the fever are produced artificially by the use of antipyrine, antifebrine and phenacetine, the crescentic and ring-shaped bodies will disappear after the administration of quinine, as quickly as the spherical bodies that are found in an ordinary case of intermittent fever. In reference to the belief that the forms of the parasite that inhabit the blood cells are not acted on by quinine, he says: "There is no doubt in my mind that this belief is not erroneous. Besides my own observations, I have been able to collect the opinions of thirty-two authors touching upon this point, and twenty-eight out of the thirty-two believe that the endo-globular or intra-corpuscular forms are not, on this account, the cause of an uncontrollable fever, and that its proximity to the blood cell does not, in any way, protect it from the action of quinine."—*St. Louis Medical Era.*

### ARISTOL IN OPERATIONS UPON THE NOSE.

Cox (*Brooklyn Med. Jour.*) says that there is no better remedy for use in the nose after operations than aristol, which "adheres closely to abraded surfaces, when insufflated, acting as a protective and cicatrizant, deodorizing most effectually without substituting any odor of its own." It is especially valuable as a dressing after operations for hypertrophy of the turbinated bones, abnormalities of the nasal septum, removal of polypi, etc.

Anderson (*N. Y. Med. Jour.*) states that when insufflated upon cut surfaces aristol acts not only as an antiseptic, but adheres firmly and prevents the constant oozing of the blood and serum from the wound. Furthermore, it reduces to a minimum any reaction occurring as a result of the operation.—*Med. News.*

---

### INSTRUCTION IN THE ADMINISTRATION OF ANESTHETICS.

At most of our hospitals the necessity has been admitted within the past few years of giving the students some instruction in the administration of anesthetics. Indeed, much evidence could be brought forward to show that the authorities of the medical schools have not as yet properly grasped the expediency of placing the instruction in anesthetization upon a proper, systematic, and complete basis. Seeing the importance of this branch of medical science, the authorities of medical schools would do well to arrange for a course of lectures upon the administration of anesthetics so that the science and practice of the subject could be properly and regularly taught. Until some improvement is effected in this regard the students can never be equipped with that measure of knowledge of the administration of anesthetics which they should possess on leaving their *alma mater*. Of course proficiency can only come from actual practice, but what we contend is that there is much more of the subject which could be taught were the opportunity to be placed in the way of the student of learning it.—*Medical Press and Circular.*

---

"Did this shooting take place in the Annex?"

"No, sir, yer Anner. It was in the small of the back, about foive or six inches above his annex."—*Ex.*

### PHYSICIANS IN THE UNITED STATES AND CANADA.

According to the Business Address Company, of New York, the following is the numerical distribution of physicians in the United States and Canada at the present time:

| | | | |
|---|---|---|---|
| Maine | 1209 | Michigan | 3957 |
| New Hampshire | 695 | Wisconsin | 1955 |
| Vermont | 694 | Minnesota | 1502 |
| Massachusetts | 4375 | Iowa | 3576 |
| Rhode Island | 561 | Nebraska | 1666 |
| Connecticut | 1398 | Kansas | 2415 |
| New York | 11378 | Arkansas | 2076 |
| New Jersey | 1842 | Missouri | 5682 |
| Pennsylvania | 8528 | California | 3515 |
| Delaware | 315 | Colorado | 1097 |
| Ohio | 7501 | Nevada | 61 |
| Indiana | 4922 | Oregon | 618 |
| Illinois | 7296 | District of Columbia | 715 |
| Maryland | 2171 | Arizona | 99 |
| Virginia | 1950 | North Dakota | 305 |
| West Virginia | 1327 | South Dakota | 578 |
| North Carolina | 1428 | Idaho | 162 |
| South Carolina | 1125 | Indian Territory | 470 |
| Georgia | 2212 | Montana | 316 |
| Florida | 801 | New Mexico Territory | 148 |
| Kentucky | 3225 | Utah Territory | 311 |
| Tennessee | 3391 | Washington | 815 |
| Alabama | 2012 | Wyoming Territory | 80 |
| Mississippi | 1520 | Oklahoma | 436 |
| Louisiana | 1476 | Alaska | 15 |
| Texas | 4866 | Canada | 4911 |

Total............... 115,779.

---

A noted London chemist has analyzed Keeley's bichloride cure with the following result: Water, 31.61 per cent.; sugar, 6 per cent.; a trace of mineral salts, principally lime; and 27.55 per cent. of strong alcohol.—*Nature.*

